# Supplementary material for: “They never appear on TV and if they have, I might have missed that moment.” How publics in South Africa and Germany view visible scientists
Source: PLoS One. 2025 Jan 29;20(1):e0316991. doi: 10.1371/journal.pone.0316991 (PMC11778643; doi:10.1371/journal.pone.0316991)
Supplement: Attachment — Submitted filename: Response to reviewers_R.docx [file pone.0316991.s002.docx]

| **Reviewer comment** | **Authors’ response** |
| --- | --- |
| **Reviewer #1** |  |
| Given that this is a cross-country study, I appreciated the section in the introduction that explained the perceptions and expectations in Germany and South Africa. In line with this, the discussion could be improved by expounding further on the implications of the results in terms of the Global North and South. Also, are there any cultural factors that should be noted? | Thank you for this important comment. In addition to the explanations in the previous version of the manuscript, we have now made it clearer (p. 5), that – while we study one country from the Global North and one country from the Global South – we do not necessarily see Germany and SA as ‘representative’ of their respective geopolitical category (although we do acknowledge the notable socio-economic differences between these two countries). As regards the cultural factors, the argument then is that our result of an overall rather similar perception of visible scientists in one Global North and one Global South country may be explained by a universal public image of science around the world, which also includes expectations towards scientists as visible scientists. This finding is confirmed beyond the two cases in another recent paper that studied 16 countries and reports rather similar characteristics for becoming a visible scientist across countries (Joubert et al. 2023). |
| What are the limitations of the study? | Thanks for this comment. We have added a paragraph on the limitations of our study on p. 31. |
| Some figures appear to be blurry. | Thank you for pointing this out. This seems to be an unfortunate effect of scaling up the figure size in the proof manuscript. The original Fig. 1a and 1b are not blurry and they should reproduce with acceptable quality if inserted at the correct (intended) size in the final proof. |
| **Reviewer #2** |  |
| Because the study compared and contrasted perceptions from 2 countries, it may be best to organize the data using tables where data coming from South Africa is on one side/column and data from Germany is on the other side/column so that similarities and differences are easier to track. The existing tables containing numbers are helpful but these can be confusing to understand especially if the reader would like to focus on qualitative information. | Thank you for this comment.  We agree that in their current form, Tab. 2 and Tab. 3 are a little difficult to read. Due to the complex nature of the correlations which we present, however, it is not possible to present this information in the comprehensive form with comparative columns as we do in Tab. 1. Nonetheless, we have changed the format of Tab. 2 and 3, and created tables for each Germany (a) and South Africa (b). To make them more reader friendly, we emphasize relevant results in bold, and there are footnotes for additional explanations. |
| It may also help if authors discuss the theoretical/practical implications of the research. Can future studies focus on the current study's limitations? How can the findings guide scientists who wish to become visible or are asked to speak in public about their research and/or findings? How do they maintain credibility given the public's perception of visible scientists? | Thanks for pointing out this important point. We have added a paragraph on the limitations of our study on p.31, which also addresses options for future study.  We have also emphasized throughout the paper the continuities in the role of visible scientists, as well as the large similarities between the two countries under study. As we show, now added on p. 27, credibility is first and foremost linked to academic reputation. At the same time, and as a practical advice to social media-active scientists, we have stressed the finding that the surveyed publics are not interested in details about the scientists’ private lives as a recommendation (p. 27/28). |
