# Supplementary material for: “They never appear on TV and if they have, I might have missed that moment.” How publics in South Africa and Germany view visible scientists
Source: PLoS One. 2025 Jan 29;20(1):e0316991. doi: 10.1371/journal.pone.0316991 (PMC11778643; doi:10.1371/journal.pone.0316991)
Supplement: Attachment — Submitted filename: Response to reviewers_R2_final.docx [file pone.0316991.s003.docx]

| **Reviewer comment** | **Authors’ response** |
| --- | --- |
| **Reviewer #2** |  |
| I appreciate the effort to address the comments. The revised manuscript is an improved version of the previous submission. The current paper presents concepts and findings in a clearer manner -- easier to understand and grasp. Authors were also better able to articulate limitations and implications. | Thank you for this acknowledgement of our revisions. |
| **Reviewer #3** |  |
| The first (weakness) concerns the fact that by asking interviewees what they think about visible scientists without distinguishing between those who have long since passed away (for example, Einstein) and those still living (and thus active), a certain confusion is introduced that may distort the responses. Obviously, this issue cannot be resolved after the survey has already been conducted, but it should be addressed with greater awareness. | Thank you for this comment.  In our questionnaire, we informed our respondents as follows:  ***Intro:*** *While there are many scientists, only some of them are well-known (visible) to the public. By this we mean that they regularly appear in the mass media including on television, in print and online news, in radio, and on social media platforms. We will refer to these scientists from now on as “visible scientists”.*  ***Open-ended****: If you can, please name up to* ***three visible living scientists*** *in your country.*  With this wording, we have tried to make it as clear as possible that we are interested in living visible scientists. Yet, as we report, our respondents still named a number of deceased visible scientists. This, for us, is a relevant result that speaks directly to our main conclusion that visibility is a rare phenomenon (p. 29). This conclusion is in line with other recent studies, e.g., Neresini et al. who state that “science and the media overlap only occasionally.” To avoid the reported confusion for future readers, we have now repeatedly explicitly stated that we asked for the names of three living visible scientists. (Abstract, Fig. 1a/b captions, and pp. 12, 14, 29 ). |
| The second weakness pertains to the connection between scientific reputation and media visibility. On one hand, the authors seem to ignore that it has been well documented how high visibility very often corresponds to low reputation (Neresini et al. 2023). On the other hand, it is obvious that people expect visible scientists to be competent and thus have a good reputation. Furthermore, the general public is rarely able to establish reputation according to the criteria of the scientific community; rather, they can infer it from media visibility (if a scientist appears in the media, then he/she must be competent). However, on this point, the conclusions reached by the authors remain almost exclusively descriptive, while a more in-depth discussion would be necessary. | Thanks for pointing out this important point. In the manuscript, we introduce scientific reputation and media prominence (visibility) as analytical concepts (p. 6ff), that can be conceptually separated while they are in practice interrelated in multiple ways, and subject to empirical study. We have included the reference to the recent low-reputation finding on p. 6 – thanks.  We agree with R#3 that it is expectable that respondents expect visible scientists to have a good scientific reputation; yet, we think it is a relevant empirical contribution that our survey shows that a good reputation is indeed the most asked-for characteristic in visible scientists. Interestingly though, and in contrast to the findings by Neresini et al, (which we now cite also in the discussion section), our respondents report that they perceive the visible scientists they can think of as having a good scientific reputation (Fig. 1a/b). Yet, we do not perceive this as a contradiction. Neresini et al. are chiefly concerned with trajectories of scientists from entering the media arena to becoming visible scientists, and they explain the characteristics of ‘media stars’ during the COVID-19 pandemic in Italy. They use the h-index as an indicator of scientific reputation to show that this indicator is less predictive than other selectors, such as the previous presence in the media, the institutional role/position, and the match between credentials and media competence.  In our study, we report the public perception of scientists in Germany and South Africa. While we find that these publics both perceive and expect visible scientists to have a good reputation, our criteria (directly derived from Goodell’s seminal study) indicate, that media-related criteria do indeed play a role in the perception of visible scientists, and thus confirm this aspect of Neresini et al.´s findings. It will be up to further (comparative) research to eventually shed light on the unsolved aspects of this research. |
